# Controlled morphology and optical properties of n-type porous silicon: effect of magnetic field and electrode-assisted LEF

**DOI:** 10.1186/1556-276X-9-512

**Published:** 2014-09-19

**Authors:** Edgar E Antunez, Jose Campos, Miguel A Basurto, Vivechana Agarwal

**Affiliations:** 1 Center for Research in Engineering and Applied Sciences, UAEM, Av. Universidad 1001, Col. Chamilpa, Cuernavaca, Morelos CP 62210, México; 2 Institute for Renewable Energy, UNAM, Priv. Xochicalco S/N, Temixco, Morelos CP 62580, México

**Keywords:** Lateral electric field, Magnetic field, Macropore, n-type, Structural gradient, Porous silicon, Photoluminescence

## Abstract

Fabrication of photoluminescent n-type porous silicon (nPS), using electrode-assisted lateral electric field accompanied with a perpendicular magnetic field, is reported. The results have been compared with the porous structures fabricated by means of conventional anodization and electrode-assisted lateral electric field without magnetic field. The lateral electric field (LEF) applied across the silicon substrate leads to the formation of structural gradient in terms of density, dimension, and depth of the etched pores. Apart from the pore shape tunability, the simultaneous application of LEF and magnetic field (MF) contributes to a reduction of the dimension of the pores and promotes relatively more defined pore tips as well as a decreased side-branching in the pore walls of the macroporous structure. Additionally, when using magnetic field-assisted etching, within a certain range of LEF, an enhancement of the photoluminescence (PL) response was obtained.

## Background

It is well known that a wide gamut of different morphologies of porous silicon (PS) can be obtained under a variety of different fabrication parameters. Generally, morphology is highly dependent on the intrinsic properties of the silicon substrate along with key fabrication parameters such as current density, hydrofluoric acid (HF) concentration, doping type, dopant concentration, and, in some cases, the illumination conditions [1]. Moreover, PS formed using p-type silicon (p-Si) or n-type silicon (n-Si) have many differences in terms of morphological characteristics (i.e., pore size, degree of branching, and orientation) [2,3]. Additionally, PS fabricated in the dark or under illumination exhibit different morphological properties [4]. Although most of the PS photonic devices are produced on p-Si, for light-emitting diode technology and microelectronic applications, n-Si is preferred. On the other hand, control of the morphology is necessary when distinct structural characteristics are required on the same chip, i.e., samples with a structural gradient (in terms of density, dimension, and depth of the pores) which are widely used in biological applications as a porous media for the culturing of biological specimens [5-8]. Conventional methodologies have been well established for n-type porous silicon (nPS) fabrication, and the preferred method requires light-assisted etching (back/frontside) to photogenerate valance band holes necessary for silicon dissolution [9]; nevertheless, it is a depth-limited process (light cannot penetrate through to the bottom layers). Hence, some methods have been proposed to fabricate photoluminescent nPS under dark conditions (without illumination): Hall effect [10] and electrode-assisted lateral electric field [11]. The former involves the application of mutually perpendicular electric and magnetic fields (within the range of 0 to 20 mT) resulting in photoluminescent nPS displaying a structural gradient in terms of thickness and light-emission properties along the lateral electric field (LEF) direction. On the other hand, the second method reports macropore formation using an electrode-assisted LEF (e-LEF) setup (30 to 50 V). Photoluminescent sample exhibiting minimum PS formation from the total effective area exposed to the HF electrolyte was obtained. However, the effects of the fabrication parameters on the resultant morphology of the sample along with the addition of a magnetic field (MF) under the e-LEF setup [11] have not been explored yet. In this work, we report on the resultant structural effect due to the simultaneous application of electric and magnetic field during the fabrication process and the morphologies thus obtained when one of those key parameters is varied. The corresponding changes in the photoluminescence (PL) properties have also been explored.

## Methods

**Figure 1:**
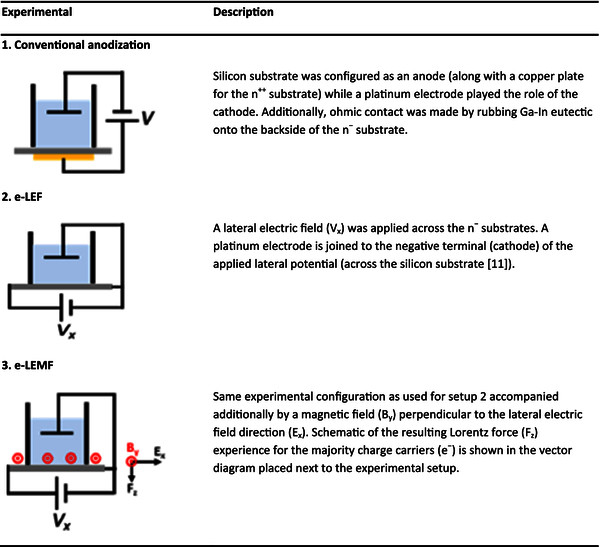
Schematic and description of the experimental setups used for the fabrication of the nPS samples.

Samples were prepared by electrochemical etching of n-Si substrates (phosphorous doped, single-side polished, (100) oriented) with an effective circular shape area of 100 mm^2^ exposed to the electrolyte. To study the effect of electric resistivity of the wafer on the morphological characteristics of nPS samples, two n-Si wafers were used: highly doped substrates (n^++^) with a resistivity range of 1 to 5 mΩ cm and low-doped substrates (n^-^) with a resistivity of 8 to 12 Ω cm. The electrolyte consisted of a mixture of aqueous 48 wt.% HF (hydrofluoric acid) and absolute ethanol (99.9%) in a volumetric ratio of 1:4, respectively. Etching was performed using a small cell made of Teflon and utilizing different experimental setups schematically represented in Figure 1. For conventional anodization (Figure 1 (1)) of n^-^ substrates, ohmic contacts were prepared by rubbing Ga-In eutectic onto the back surface of the substrate while for setups e-LEF (Figure 1 (2)) and electrode-assisted lateral electric and perpendicular magnetic field (e-LEMF) (Figure 1 (3)), Ga-In eutectic was rubbed only at the two extreme ends for each substrate. Nomenclature of cathode and anode terminals will be used from now on to name the locations (on the etched area of the sample) close to the contacts where the positive and negative terminals of the LEF were applied to the substrate (Figure 1 (2) and (3), respectively). Etching process was executed under dark conditions (no illumination) and at room temperature. PL response was obtained using a Cary Eclipse Fluorescence Spectrophotometer (Varian Inc., Palo Alto, CA, USA) by fixing a 250 nm excitation wavelength collecting the spectra close to the anodic region for each of the samples along the LEF direction. The morphologies of the etched porous layers (top and cross-sectional views) were observed using two different scanning electron microscopes FESEM Hitachi S5500 and SEM Hitachi SU1510 (Hitachi High Technologies Canada, Inc., Toronto, Canada).

## Results and discussion

**Figure 2:**
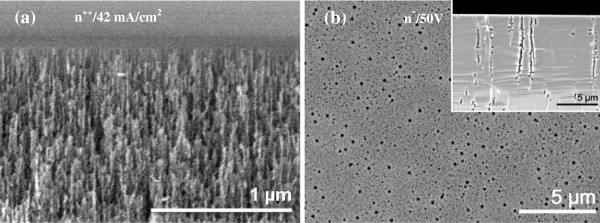
**Micrographs of nPS samples fabricated using conventional anodization. (a)** n^++^ substrate (42 mA cm^**-**2^, 65 s) and **(b)** n^-^ substrate (50 V for 30 min). Cross-sectional view of circular macropores of the n^-^ sample is shown in the corresponding inset.

In order to investigate the influence of fabrication parameters on the structural characteristics of the nPS samples, three parameters were studied: (a) resistivity of the n-Si wafer, (b) LEF, and (c) MF. Substrates of two different resistivities in the range of 1 to 5 mΩ cm and 8 to 12 Ω cm where etched using the conventional anodization setup, resulting in two different pore morphologies. As it is well known, nanometer-scale features (i.e., micro- and mesopores) were obtained when a current density of 42 mA cm^-2^ was applied to the n^++^ substrate during 65 s. Figure 2a shows the mesoporous layer (thickness >1 μm) containing pores with average diameter of 45 nm, approximately. Conversely, when a potential of 50 V during 30 min was applied to the n^-^ substrate, much less dissolution of silicon (under dark conditions) resulted in the formation of circular macropores with an average diameter of 230 nm as shown in Figure 2b. Cross-sectional view of some circular macropores formed on the n^-^ sample displaying bottleneck features at the opening of the pores as well as the presence of side-branching along the pore growth direction are shown in the inset of Figure 2b.

**Figure 3:**
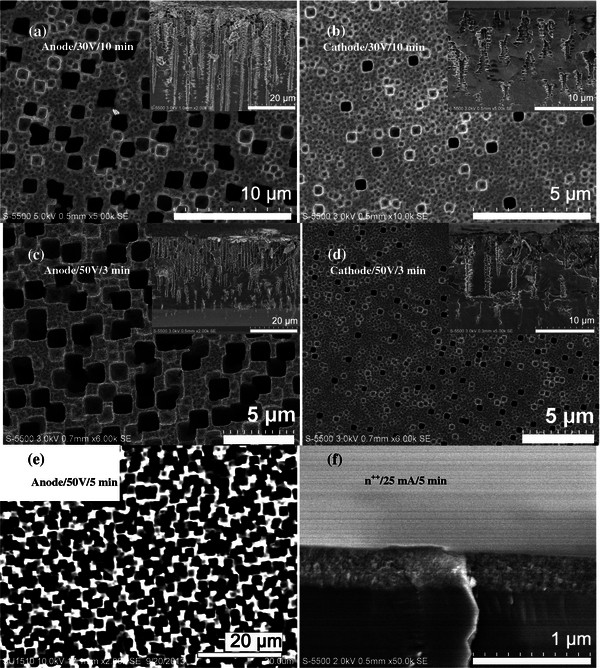
**Top and cross-sectional micrographs of nPS samples fabricated using e-LEF setup.** Effect of LEF at locations close to the anode and cathode: **(a-b)** 30 V for 10 min and **(c-d)** 50 V for 3 min. **(e)** Top image close to the anodic region of the sample etched at 50 V during 5 min and **(f)** cross-sectional view of the n^++^ sample etched applying a lateral current of 25 mA for 5 min.

To study the effect of MF when using the e-LEF setup, two control samples were fabricated in the absence of MF (Figure 1 (2)), i.e., n-Si wafers were etched under the influence of low and high LEFs (30 V and 50 V, respectively) applied across the substrates. Top and cross-sectional micrographs of nPS samples fabricated using the e-LEF setup are shown in Figure 3a, b, c, d, e, f. Figure 3a, b shows top-view micrographs taken at locations close to the anode and cathode terminals of the etched samples formed when a constant LEF of 30 V was applied to the n^-^ substrate during 10 min. Cross-sectional views are shown in the insets of Figure 3a, b. The resultant porous layer exhibited large square-shaped macropores (width of approximately 1.3 μm) with a thickness up to 40 μm close to the anode location, while an evident decrease in the pore width (approximately 500 nm) and thickness (approximately 15 μm) of the porous film was observed towards the cathode location of the sample (along the LEF direction). Side-branching appears as an essential structural feature of the macropore formation in low-doped Si wafers (side-branching is formed due to avalanche breakdown effect). On the other hand, to investigate the effect of higher LEF, a constant lateral potential of 50 V was applied across the n-Si samples for 3 min. Figure 3c, d shows the top and cross-sectional views of the sample close to the anode and cathode ends, respectively. Formation of square-shaped macropores (average width of 1.5 μm) and the presence of a structural gradient, i.e., denser and larger macropores, were found close to the anodic region while a decreased number of macropores with reduced dimensions (approximately 500 nm) were observed close to the cathode. Insets of Figure 3c, d reveal the cross-sectional view of the sample with a thickness of approximately 30 μm close to the anode and an apparent diminishing of the side-branching as compared with the sample fabricated using low LEF (30 V). However, an increased amount of nucleation sites on the effective etched surface of the sample was observed which gives rise to the fact that if an extended etching time is employed, when higher lateral potentials are applied, the dominance of overlapped square-shaped macropore formation will take place, thus increasing the porosity of the sample (voids). It is important to note that any further increase in the magnitude of LEF (>50 V) resulted in the heating and evaporation of the HF electrolyte, even for short etching times. On the other hand, lower LEF (<30 V) required longer etching times (>15 min) in order to form observable macroporous features which appeared in less density over the complete sample.

**Figure 4:**
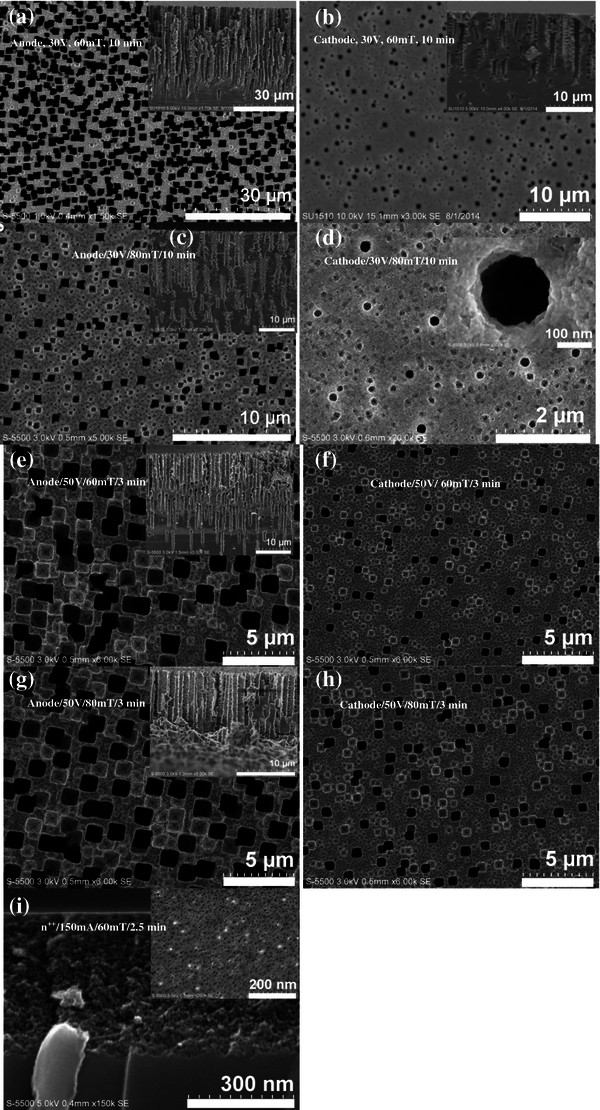
**Top and cross-sectional micrographs of nPS samples fabricated using e-LEMF setup.** Effect of MF at locations close the anode and cathode: **(a-b)** 30 V and 60 mT for 10 min, **(c-d)** 30 V and 80 mT for 10 min, **(e-f)** 50 V and 60 mT for 3 min, **(g-h)** 50 V and 80 mT for 3 min, and **(i)** cross-sectional and top image of the n^++^ sample etched applying a lateral current of 150 mA and 60 mT for 2.5 min.

Figure 3e shows the top view (SEM image) of n^-^ sample fabricated applying a high LEF of 50 V and an increased etching time of 5 min (half of the etching time used when applying a low LEF of 30 V). The large amount of nucleation sites leads to the formation of larger square-shaped macropores causing a decrease in the main pore-to-pore distance, thus resulting in an overlapping in the opening of pores, which eventually will cause the collapse of the PS layer close to the anodic region. Finally, to observe the morphology effect due to the electrical resistivity of the wafer, a n^++^ sample (1 to 5 mΩ cm) was etched using e-LEF setup under galvanostatic conditions with a flow of a lateral current of 25 mA for 5 min; a porous film exhibiting a sponge-like structure (composed mostly of microporous features) and a thickness of approximately 300 nm was formed (Figure 3f). In our research, highly doped substrates (n^++^) were not further studied due to the fact that the resultant structural characteristics showed no significant changes as a function of the different magnetic fields applied. The morphology obtained in all the cases was a sponge-like structure as above mentioned irrespective of the lateral current used during the fabrication process.Finally, to investigate the morphology of nPS samples under the combined effect of LEF and perpendicular MF (using the e-LEMF setup, Figure 1 (3)), various nPS samples were fabricated varying the magnitude of the MF. Figure 4a, b shows the top and cross-sectional images taken close to the anode/cathode of the nPS sample fabricated with a LEF of 30 V and a MF of 60 mT (etching time of 10 min). Large square-shaped macropores of approximately 1.7-μm width and a thickness of the porous layer up to 40 μm were obtained close to the anode while formation of reduced square-shaped macropores and decrease in the thickness (<15 μm) were observed close to the cathodic region. Similarly, Figure 4c shows the top and cross-sectional view of the nPS sample (close to the anode) fabricated by applying a lateral EF of 30 V accompanied with an increased perpendicular MF of 80 mT (etching time of 10 min). A visible reduction in terms of quantity and dimension of large square-shaped macropores (width of approximately 750 nm) with depth up to 35 μm was found, as compared with the sample fabricated with a MF of 60 mT. However, significant structural changes were observed in the anodic region of the sample when the perpendicular MF was increased. Under the above-mentioned fabrication parameters, a transition in the shape of the pores occurs from a square-like to a round-shaped, accompanied with a decrease in the dimension of the pores (≤200-nm width) as shown in Figure 4d. The inset of Figure 4d shows the presence of only round-shaped macropores at the location close to the cathode. Therefore, it can be concluded that an optimum combined effect of LEF (30 V) and MF (80 mT) results in the possible tuning of the pore dimensions as well as in a transition of the morphology of the macropores formed across the structural gradient along the LEF direction.

To study the effect of an increased lateral EF, a lateral potential of 50 V was also tested across a n-Si substrate accompanied by two different MFs (60 and 80 mT). Figure 4e, f shows the top and cross-sectional micrographs of nPS fabricated with a LEF of 50 V and a perpendicular MF of 60 mT (etching time of 3 min). Similar to the above-mentioned results, square-shaped macropores (approximately 1.5-μm width) were formed with a notably less-interconnected pore-to-pore distance (as compared with the sample prepared using the e-LEF, LEF of 50 V without MF; Figure 3c, d. Reduced square-shaped macropores of 600-nm average width were found close to the cathodic region due to the structural gradient caused by the application of LEF (50 V). However, presence of a higher MF of 80 mT during the fabrication process leads to a reduction in the width of the large square-shaped pores (approximately 1.2 μm) close to the anode location while the dimension of the pores reduces close to the cathodic region (750 nm), as shown in Figure 4g, h, respectively. Furthermore, the latter result showed a minimum pore size variation (of approximately 450 nm) in the reduction of the main width of the macropores (from 1.2 μm to 750 nm) formed along the LEF direction. Highly doped n-Si substrates under the e-LEMF configuration also resulted in a sponge-like structure (composed mostly of micro- and mesoporous features) irrespective of the lateral current and/or magnetic field (within 0 to 80 mT range) used during the fabrication process. Figure 4i shows the cross-sectional micrograph of the sample fabricated under a galvanostatic regime, a lateral current of 150 mA biased across the n^++^ sample accompanied with a MF of 60 mT (for 2.5 min). Sponge-like porous film of approximately 345 nm thick was obtained with the above-mentioned experimental conditions; inset of Figure 4i shows a top view image of the n^++^ sample. As the structural changes are governed by valence band holes available at the etching interface for the reaction, higher magnetic fields (close to 1 T) should be considered in order to observe significant structural changes using n^++^ substrates.

**Figure 5:**
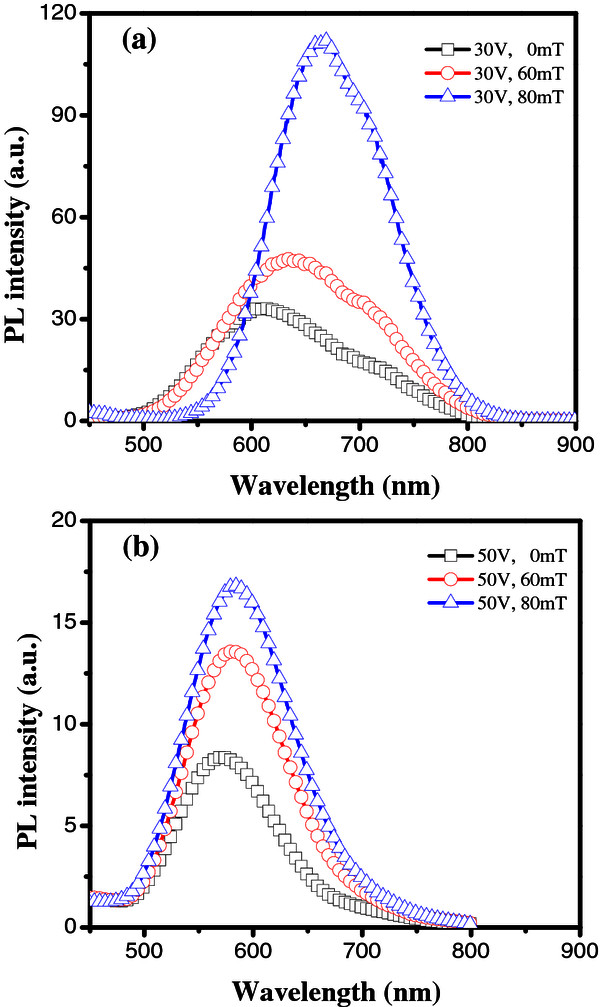
**PL spectra of nPS samples.** PL spectra taken from the anodic region of nPS samples fabricated under the combined effect of LEF and different values of MF **(a)** 30 V and **(b)** 50 V.

As the etching in n-Si wafer depends on the concentration of valance band holes available at the Si-electrolyte interface, the maximum formation of the PS layer is achieved towards the anodic location of the sample. Hence, all the PL measurements were performed at this region, with an excitation wavelength of 250 nm, to qualitatively analyze the effect. PL spectra of freshly etched nPS samples fabricated using an electrode-assisted LEF of 30 V as a function of different values of MF are presented in Figure 5a. As the PL signal from PS has been attributed to the joint contribution of quantum confinement and the surface states [12], dominant PL contribution can be attributed to the microporous film (pore dimension <2 nm) prevailing on the entire PS surface (at the top of the macropores) including the walls of the macropores as well. An increase in the MF (30 V, 80 mT) leads to a decrease in FWHM, an enhancement of the PL peak intensity, and a redshift of the PL peak wavelength. Enhancement of the maximum PL intensity can be thought as a function of the presence of the microporous structure over the sample's surface due to a decrease of the density related to the large macropores (refer to Figures 3a and 4c). Thus, drastic reduction in the average pore size and less amount of macropore formation increases the presence of micro- and mesoporous structure (small size of crystalline features associated with the quantum confinement effect) on the surface of the sample, which is responsible for PL emission. The observed redshift in the PL peak wavelength from the sample fabricated using 80 mT is possibly due to relatively large silicon crystallites formed within the microporous layer at the top of the sample's surface.On the other hand, Figure 5b presents the PL spectra of nPS samples prepared using the e-LEF of 50 V as a function of different values of MF (0, 60, and 80 mT). Irrespective of the applied magnetic field, PL spectra show a spectral bandwidth of approximately 250 nm (500 to 750 nm) and a maximum PL intensity peak around 582 nm. The enhancement of the PL intensity with an increasing magnitude of magnetic field is attributed to the increased availability of valence band holes on the silicon surface. With an increase in the MF, the density as well as the dimension of the macropores decreases and is accompanied by a relative increase in the microporous features. The presence of more number of microporous features, responsible for the quantum confinement effect, leads to an increased PL emission.

## Conclusions

Structural gradient formation (i.e., density, dimension, and depth of the pores) is highly dependent on the LEF applied across the n-type silicon substrates. At a high lateral potential (50 V), major density of pores is obtained all over the sample as compared with the PS obtained with 30 V. As compared to the other reports [11] using an e-LEF setup, demonstrating the formation of PS only at the anodic region of the total effective area of the sample, our results achieved to form PS in the complete area exposed to the electrolyte, thus enhancing the optical properties of the samples. The combined effect of high LEF (50 V) and a perpendicular magnetic field is majorly observed as a relative reduction in side-branching and tapering ends of the macropores. Under particular fabrication parameters involving the joint contribution of a LEF of 30 V and a MF of 80 mT, a structural transition from square-to-round-shaped macropores appears close to the cathodic region of the sample, opening the possibility of tuning the structural properties of the PS structure. Enhancement of the PL response was achieved by using an increased MF during the fabrication process.

## Competing interests

The authors declare that they have no competing interests.

## Authors’ contributions

EEA carried out all the experimental work. JC helped in taking the SEM images. MB helped in making the electronics more suitable for starting the automatized electrochemical fabrication process. VA and EEA conceived the experiments and finalized the manuscript. All the authors have read and approved the manuscript.

## Authors’ information

E.E.A. is a third year PhD student at the Research Center of Autonomous State University of Morelos, Mexico (CIICAp-UAEM). J.C. is a senior technician at the Energy Research Institute of National Autonomous University of Mexico (UNAM). M.B. is a scientist at CIICAp and UAEM. V.A. is a senior scientist working in the field of porous silicon and its applications at CIICAp and UAEM.
